# Exploring seed characteristics and performance through advanced physico-chemical techniques

**DOI:** 10.1038/s41598-024-75236-0

**Published:** 2024-10-15

**Authors:** Dhanalakshmi Vadivel, Rania Djemal, Jessica García, Andrea Pagano, Rahma Trabelsi, Maroua Gdoura-Ben Amor, Safa Charfeddine, Siwar Ghanmi, Ibtisem Khalifa, Mariem Rekik, Fatma Amor, Chantal Ebel, Radhouane Gdoura, Amine Elleuch, Alma Balestrazzi, Anca Macovei, Moez Hanin, Daniele Dondi

**Affiliations:** 1 Department of Chemistry, University of Pavia, Viale Taramelli 12, Pavia, 27100 Italy; 2 Plant Physiology and Functional Genomics Research Unit, Institute of Biotechnology of Sfax, University of Sfax, BP “1175”, Sfax, Tunisia; 3 Department of Biology and Biotechnology ‘L. Spallanzani’, University of Pavia, Via Ferrata 9, Pavia, 27100 Italy; 4 Research Laboratory of Environmental Toxicology Microbiology and Health (LR17ES06), Faculty of Sciences of Sfax, University of Sfax, BP 1171, Sfax, 3000 Tunisia; 5 Natural Resources, Agriculture and Environment Laboratory of Plant Biotechnology, Faculty of Science of Sfax, NCP (National Contact Point) for Horizon Europe Cl6 Food, B.P.1171, Bioeconomy, Sfax, 3000 Tunisia

**Keywords:** Seed, Physico-chemical profile, Thermogravimetric analysis, Electron paramagnetic resonance, High-performance liquid chromatography, Principal component analysis, 0-omics, Chemical biology, Computational biology and bioinformatics, Plant sciences, Chemistry

## Abstract

Simple physico-chemical techniques can be used to evaluate the composition, structure, and characteristics of plant seeds to determine their viability, quality, and possible uses in agriculture. Advanced analytical techniques, including thermo gravimetric analysis (TGA), electron paramagnetic resonance (EPR), and high-pressure liquid chromatography (HPLC), provide completely new insights and more precise information. They can be integrated to build up seed quality profiles, with great advantage to assess water content, organic compounds, and inorganic metals without the need to carry out many extraction procedures, as requested by more conventional methods. In this study, seed lots from three different plant species such as *Triticum turgidum L. subsp. durum* (wheat), *Trigonella foenum graecum* L. (trigonella or fenugreek), and *Atriplex halimus* L. (saltbush or sea orach) have been used to test the potential of TGA, EPR, and HPLC to discriminate between seed-specific features. A key finding of this study is that HPLC is essential in Principal Component Analysis (PCA) because various seeds (from the same species or other species) may contain compounds with varying polarity groups. The reported data confirm the efficacy of this approach. These data, fully available for other users, are coherently constructed and provide a proof of concept for future seed quality control studies.

**Supplementary Information:**

The online version contains supplementary material available at 10.1038/s41598-024-75236-0.

## Introduction

Plant breeders need high-quality seeds, in terms of germination performance and nutritional value, that might be used to build new varieties with unique characteristics and robust resilience to environmental stresses^1,2^. This is accomplished by carefully choosing the best individual characteristics. Often, only a minor fraction of seeds harbor the desirable traits within seed bulk samples and this can delay the assessment of the desired seed traits. Therefore, researchers’ efforts towards the production of next-generation, climate-smart crops can be significantly improved by monitoring the quality of individual seeds^1^. To speed breeding towards novel climate-resilient varieties, seed quality issues should be addressed using a multidisciplinary approach that includes chemical-physical techniques combined with molecular and biochemical tools^3^.

High-throughput multi-omics methods allow us to gain insights into gene functions, not only in terms of expression analysis (transcriptomics and proteomics), but also through the quantification of other key molecules (metabolomics), as well as the study of diversity in shape and behavior (phenomics). Several reports highlight the complex networks of cellular and molecular events underlying seed germination under standard and stress conditions, revealed by transcriptomic and proteomic analyses^4,5^. Among the different omics tools utilized to decipher the key determinants of seed quality, metabolomics is frequently used to correlate specific phenotypes to specific metabolic seed profiles^6^. It can also offer direct evidence on the metabolites (e.g. antioxidant compounds and stress protectants) contributing to stress response in seeds, hence preventing their deterioration^7–9^. Facilities have been developed to speed up omics-based investigations, as in the case of WheatOmics 1.0 and WheatGmap web-based platforms that support implementing functional genomics and computational studies in wheat^5,10,11^.

Omics-based techniques often require expensive, laborious, and time-consuming procedures^12^. The extraction protocols can be complex, particularly for seeds, well-known recalcitrant samples with high polysaccharide and polyphenol contents. Furthermore, the data analysis workflow requires multiple analytic tools to discover meaningful insights. Scalability is a main issue when merging omics datasets^13^. Data processing, storage, and analysis grow more challenging as the volume of omics data generated rises. For instance, a large-scale proteomic investigation may produce terabytes of data, but a single genomic sequence for a crop plant would need hundreds of gigabytes of storage^13^. It is evident that omics-based techniques cannot be applied on a large scale for the evaluation of high numbers of seed lots or the analysis at the single-seed level, in a cost-effective way. In this respect, the potential and efficacy of analytical methodologies (based e.g. on the evaluation of chemical-physical parameters), as tools to assess seed quality should be promoted. These assays, able to reveal fundamental organic molecules, inorganic metals, and compound absorbance, should be revisited, from a holistic standpoint, and used to set a novel methodology for the assessment of seed quality features, more suitable to companies’ requirements for large-scale quality screening of huge numbers of seed lots.

In this study, the fundamental analytical techniques TGA^14^, EPR^15^, and HPLC-UV^16^ were applied as tools to investigate the quality features of seeds from three distinct plant species: the cereal *Triticum turgidum L. subsp. durum* (wheat), the legume *Trigonella foenum graecum* (trigonella or fenugreek) and the halophytic perennial shrub *Atriplex halimus*(saltbush or sea orach). Previous reports have underlined the potential of these approaches in deciphering the complex response of seeds in terms of viability and deterioration. Gianella et al^14^.. , investigated the intra-species variation of eight *Pisum sativum*L. accessions using TGA measurements that allowed to track the dynamics of samples’ breakdown during heating, identify the various reaction stages as a function of temperature, and finally provide seed-specific percentages of decomposition^14^. TGA has been applied to seeds of other plant species such as date palm (*Phoenix dactylifera*L.)^17,18^, watermelon (*Citrullus lanatus*L.)^19^, and neem (*Azadirachta indica*L.)^20^.

**Scheme 1:**
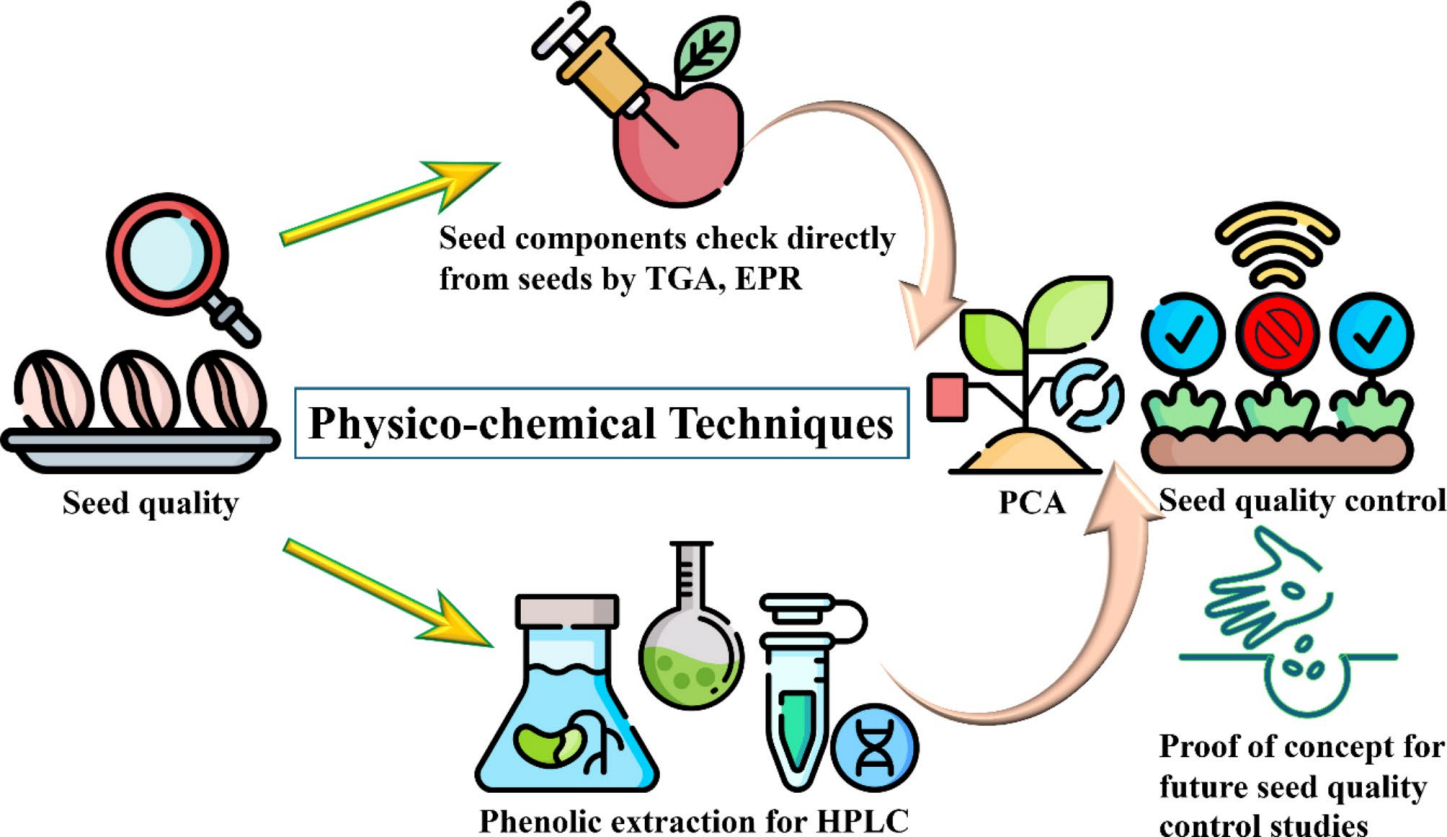
Schematic illustrating the physico-chemical method for analyzing seed properties.

The free radical profiles in seeds and their relationship to germination rates were observed using EPR spectroscopy^15^. These studies emphasize the -irradiation impact on seed storage that clarifies the connection between ROS buildup, γ-irradiation, and seed quality in four native alpine species^15^. EPR proved useful to address seed quality-related issues such as pea seed response to aging^14^, and the impact of oxidative damage on *Medicago truncatula*L. seeds subjected to rehydration-dehydration cycles^4^, hormopriming^8^, and to genotoxic agents^7^. Reports concerning HPLC applied to seeds cover a wide range of secondary metabolites with relevance in terms of nutraceutical and pharmacological value, such as flavonoids^21,22^. EPR, TGA, and HPLC-UV measurements performed on seeds of the three plant species allowed us to gather information on carbon radicals with metals, organic polymers, and metabolite composition, respectively as shown in Scheme 1. Principal Component Analysis (PCA) integrating the results from EPR, TGA, and HPLC-UV analyses and seed germination data were elaborated and consolidated with the GNU Octave program, allowing a 3D view representative of data relative distribution and origin. The program hereby developed is available to researchers and the investigations are coherently constructed providing a proof of concept for future studies on seed physiology, metabolism, and quality.

## Experimental part

### Plant materials

The seed lots used in this study were provided by the Institut National des Grandes Cultures (INGC, Tunisia). The list of seed lots is shown in Table 1. Four different wheat genotypes along with two trigonella and one *Atriplex* genotype, were evaluated.

**Table 1:** Seed lots were used in this study.

Seed code	Species/genotype
A	*Triticum turgidum L. subsp. durum* /Mahmoudi
B	*Triticum turgidum L. subsp. durum* /Maali
C	*Triticum turgidum L. subsp. durum* /Karim
D	*Triticum turgidum L. subsp. durum* /Jnah Khotifa
E	*Atriplex halimus* L.
F	*Trigonella foenum graecum* L./Ryhane
G	*Trigonella foenum graecum* L./Tborsek

## Thermogravimetric analysis

Thermogravimetric analyses were performed using a Mettler Toledo (Switzerland) TGA1 XP1 thermogravimetric analyzer^23^. Each sample was subjected to a crushing process until obtaining a powder texture. Samples weighing 8–10 mg were inserted in an alumina crucible with a perforated lid at the temperature from 25 °C to 800 °C by the heating rate of 10 °C/min under nitrogen with a flow rate of 100 mL/min. A blank performed in the same heating conditions with an empty crucible together with a perforated lid was subtracted from the curves.

## Electron paramagnetic resonance

The EPR instrument used for the measurements was a Bruker (US) EMX, X-band continuous wave spectrometer equipped with an EPR cavity Bruker ER4119HS^24^. Modulation amplitude: 0.5 G, microwave power: 1 or 10 mW, 5 scans. For EPR analysis, samples did not undergo any treatment. Seeds were introduced directly in the EPR quartz tube.

## High-pressure liquid chromatographic measurement

High-pressure liquid chromatographic analyses were carried out on a Waters (US) HPLC instrument equipped with a Waters 1515 pump, autosampler Waters 2707 and a UV-Vis detector Waters 2487^25^, with C-18 reverse phase Phenomenex column (150 × 4.6 mm, particle size 5 μm); the samples were eluted with an isocratic phase consisting of A: 0.1% acetic acid in water/B: acetonitrile (70:30 v/v). The injection volume was 5 µL, the column temperature was set at 30 °C, the flow rate was 1 mL/min; the chromatograms were recorded at the wavelength of 254 nm. Seeds are crushed and extracted as in the reported procedure^26^.

## Principal component analysis

For PCA analysis and the automatic generation of the figures included in this paper in the principal component analysis section, an Octave code was used. The code is provided in the supplementary information file for free use.

## Results and discussion

### Thermogravimetric analysis (TGA) of seeds

**Fig. 1:**
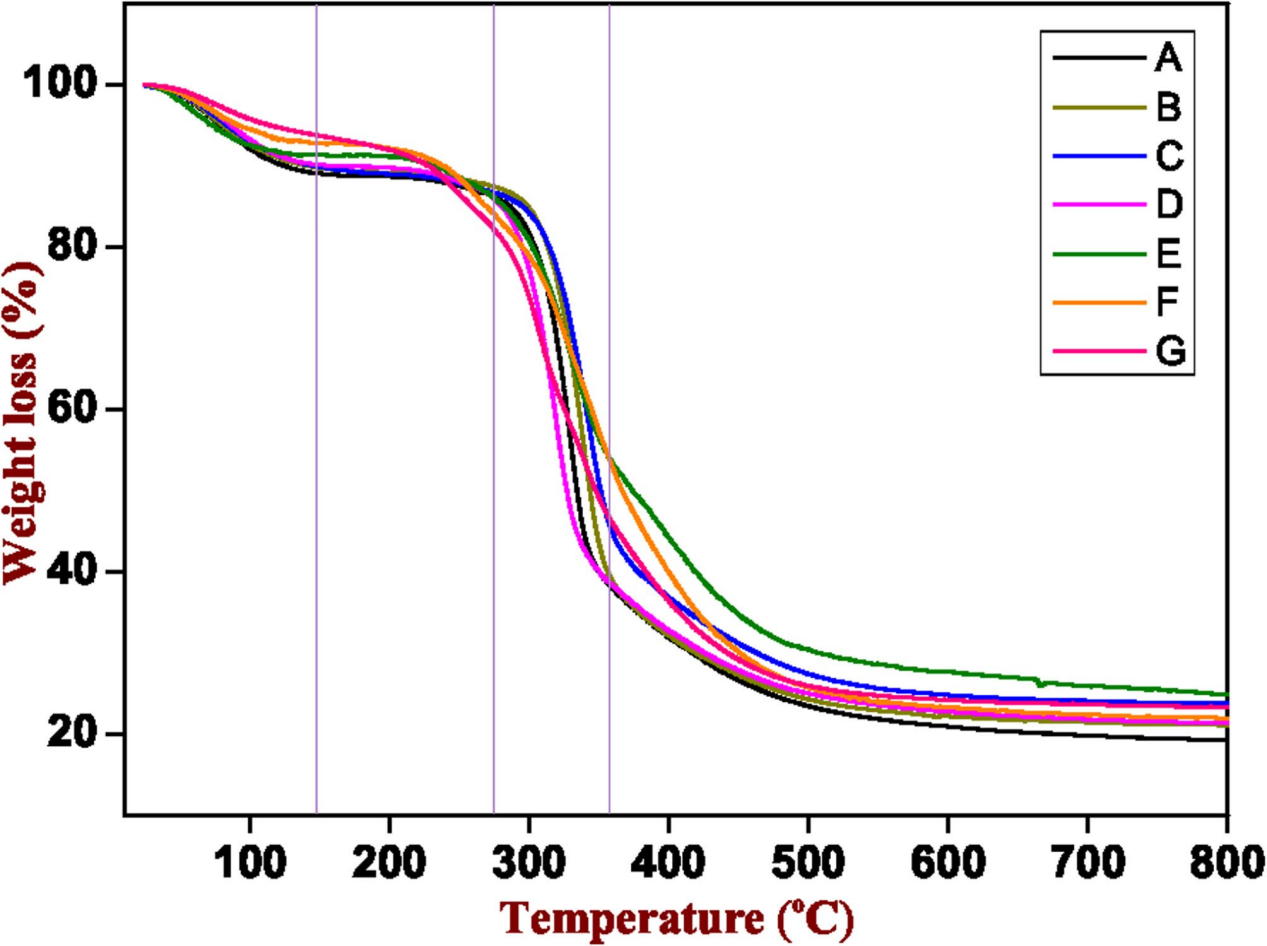
TGA thermogram of seeds from different genotypes/species obtained under nitrogen atmosphere. Seed code from (A-G) is coherent with Table 1.

TGA was used to investigate and compare the biochemical composition of each different seed type (cereal, legume, halophyte). The analysis was performed on a sample obtained by grinding four seeds in a mortar to produce a uniform powder. Different weight losses are visible, depending on the temperature used during the TGA. Similar curve profiles were obtained with all the tested seeds (Fig. 1). This finding suggests that seeds from phylogenetically distant plant species share similar TGA thermograms and, thus, this feature alone cannot be used to distinguish between seeds of different species.

As reported in Fig. 1, the first thermal decomposition occurs around 150 °C showing the weight loss corresponding to the seed moisture content. The second region of thermal decomposition is around 150–275 °C, showing the weight loss corresponding to the oils and volatile matter. The thermal decomposition range around 275–350 °C corresponds to the decomposition of cellulose or starch polymers. Finally, the weight loss above 350 °C corresponds to the presence of chitin-type polymers^27,28^. The values obtained from TGA and used for PCA are listed in Table 2 with the following labels: TGA 25–150 °C, TGA 150–275 °C, TGA 275–350 °C, and TGA 350–800 °C. Data analysis was carried out on each tested seed type about % weight loss and temperature, selecting the intervals that are key points of degradation present in the sample. The differential thermal analysis (DTA) is shown in Figure S1.

**Table 2:** Average weight losses obtained from TGA analysis performed on 5 different seeds for each species/genotype with standard deviation. The weight loss percentage indicated in the temperature range specified by columns concerning seeds A-G is coherent with Table 1.

Seed code	25–150 °C	150–275 °C	275–350 °C	350–800 °C
A	−7.6 ± 2	−2.8 ± 0.7	−50.8 ± 5	−20.2 ± 3.7
B	−8.0 ± 0.7	−3.4 ± 1	−45.2 ± 8	−21.6 ± 6
C	−8.9 ± 0.8	−2.9 ± 0.9	−44.2 ± 8	−25.4 ± 5.0
D	−8.0 ± 1.3	−3.2 ± 1.1	−48.3 ± 4	−20.2 ± 2.7
E	−7.4 ± 1.8	−7.4 ± 1.8	−28.1 ± 3	−32.3 ± 3
F	−7.1 ± 1	−9.3 ± 1.8	−27.1 ± 4	−36.2 ± 2.7
G	−5.4 ± 1	−10 ± 2.2	−33 ± 1.9	−29.3 ± 3

## Electron Paramagnetic Resonance of seeds

**Fig. 2:**
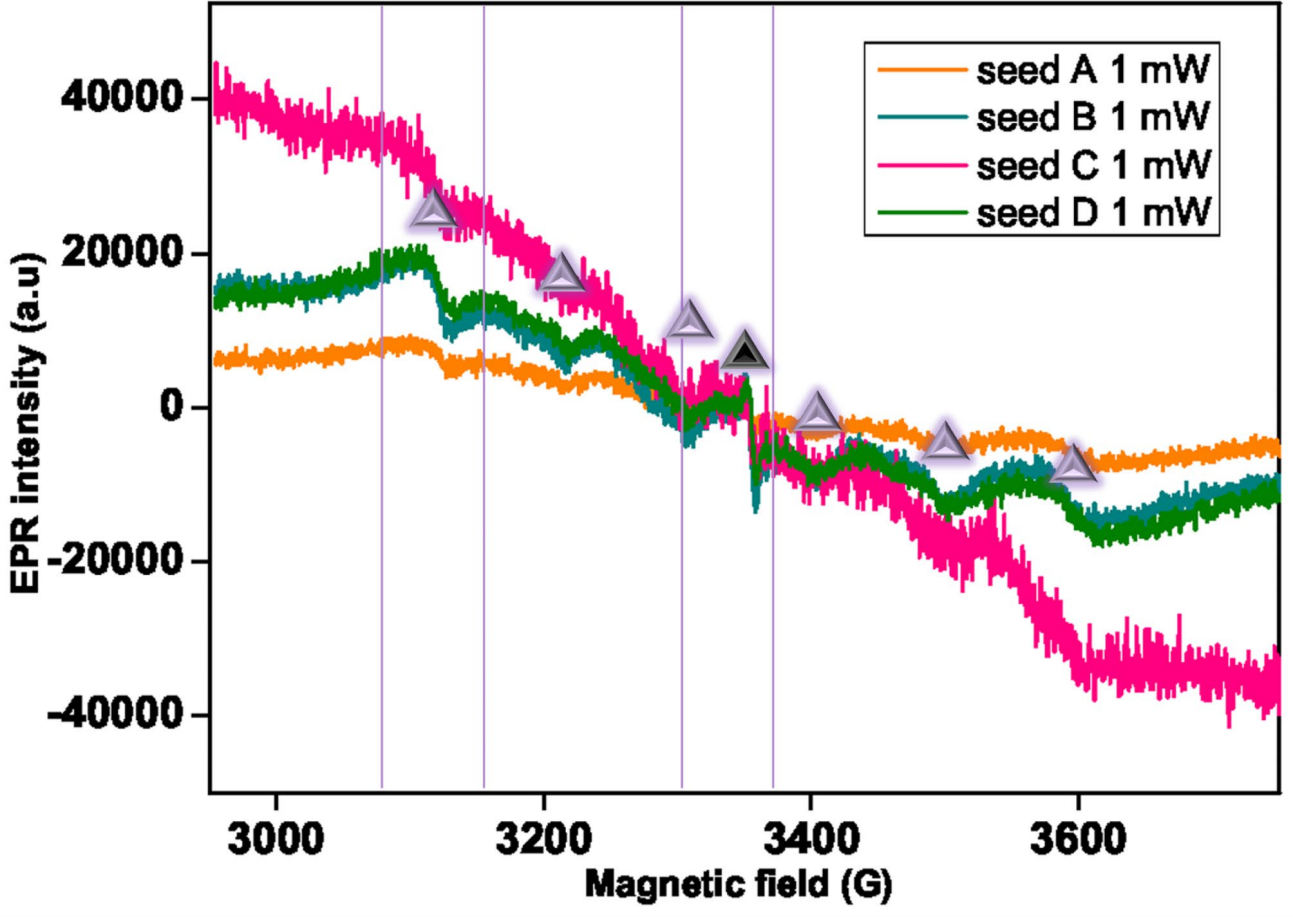
EPR spectra of seeds belonging to the wheat (*T. durum* L.) genotypes Mahmoudi (A), Maali (B), Karim (C), and Jnah Khotifa (D) were obtained by measuring with the microwave power of 1 mW at room temperature.

The EPR spectra of the wheat (A, B, C, and D) seeds are displayed in Fig. 2. Under the measurement condition of 1 mW, the magnetic field (G) around 3350 G value indicates the presence of carbon radicals^24,29,30^which originate from proteins or other organic compounds. Wheat seeds were characterized by macroelement magnesium, whereas manganese, iron, copper, zinc, chromium, and nickel^31^ are among the microelements that are often present. The five unpaired electrons in manganese divide the spectral line into six (broad) because of its spin of 5/2 which is shown in Fig. 2. As shown in the literature, organic moieties, and manganese (II) metal may be detected interacting in the 3310–3410 G range^8^.

As shown in Figure S2, the EPR spectrum was recorded at 10 mW. Since organic molecules have longer relaxation times concerning metals, the signal around 3350 G (corresponding to the carbon radicals) decreases and is dominated by the metals.

**Fig. 3:**
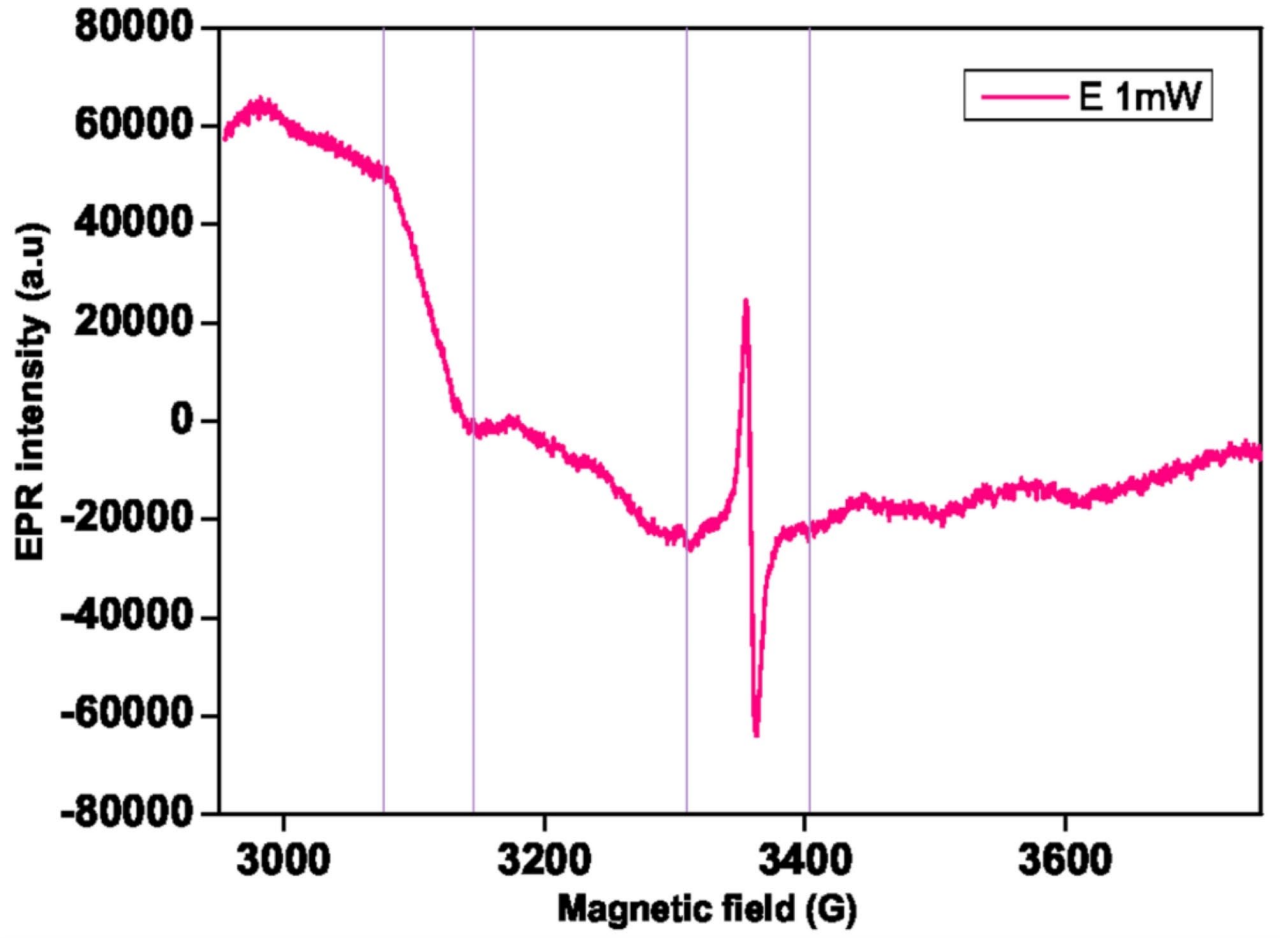
EPR spectra of seeds belonging to *A. halimus* L. were measured with the microwave power of 1 mW at room temperature.

The magnetic field (G) around 3350 G value in the EPR spectra of the seed E (*Atriplex*) shown in Fig. 3under 1 mW measurement conditions confirms the presence of carbon radical (as in seed A-D) with a sharpened peak. This is because there is a slightly higher presence of carbon radicals when compared to other species like A-D. The background of the spectrum is affected by the iron ion influence that is observed in the elevation of the peak around 3100 G. This effect either mask the presence of manganese ions or might indicate a lower quantity. Furthermore, an iron metal impact can be seen in the EPR because, when the measurement power is increased to 10 mW, the peak broadens and changes substantially below 3100 G (Figure S3)^32^.

**Fig. 4:**
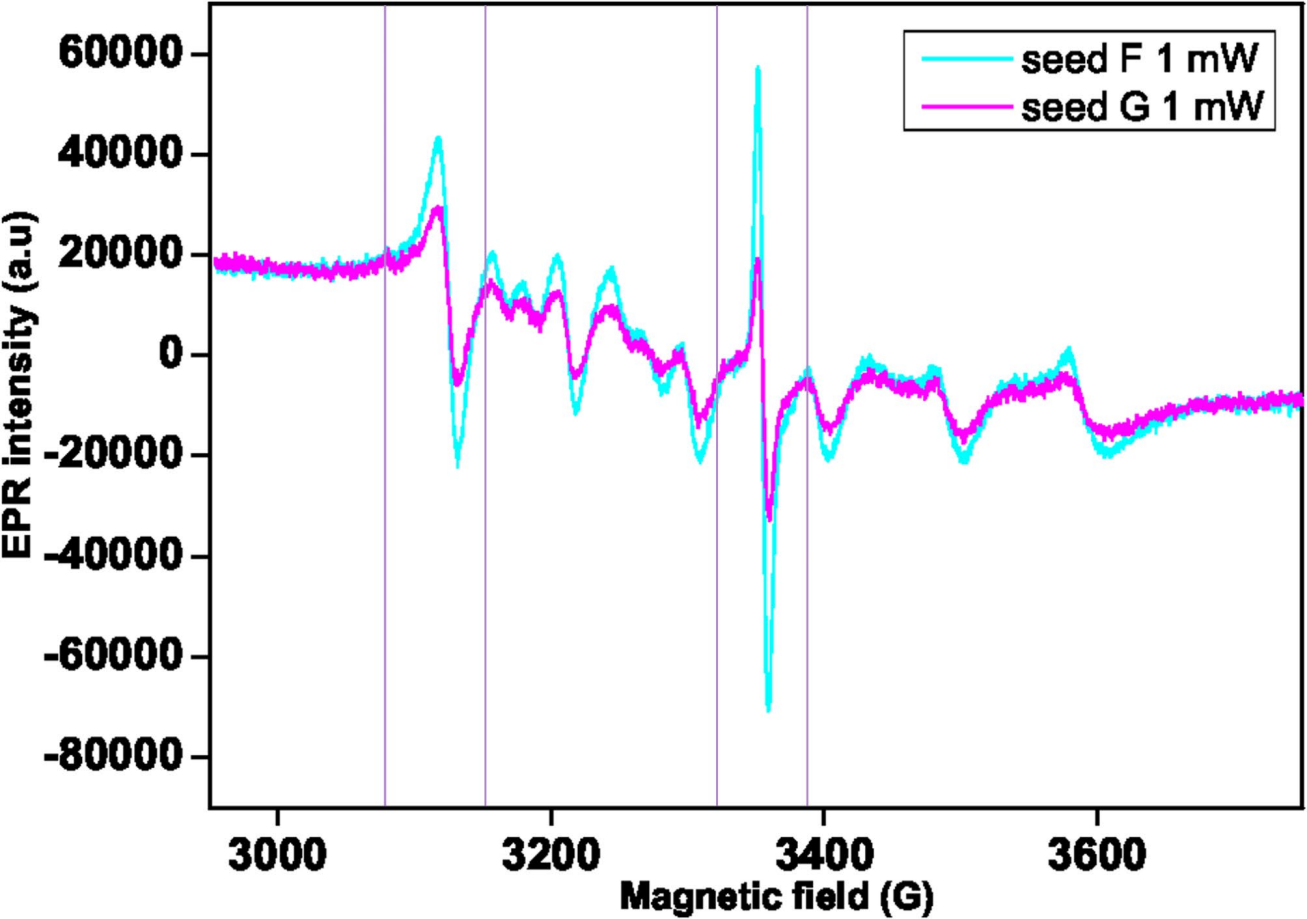
EPR spectra of the seeds belonging to the trigonella (*T. foenum graecum* L.) genotypes F and G were measured with the microwave power of 1 mW at room temperature.

For the seeds F and G (Fenugreek), the EPR spectra were observed under 1 mW overlay and show at G ~ 3350 a footprint for carbon radicals like the one observed with *Atriplex* (Fig. 4). However, these spectra show other significant differences, and the signal splitting at a G value of 3100, indicates the presence of copper and manganese metals in the seed^33^. Figure S4 illustrates the increased energy dispersion of metals that dominates the carbon radical peaks (organic polymers/compounds) under 10 mW measurement conditions. Moreover, the change in the background of the spectral line indicates the existence of iron metal^33^.

Each of the following varieties (A, B, C, D, E, F, and G) were measured. We took into consideration the region of manganese and copper (3100 G, EPR M (metal region)) and carbon radicals (3350 G, EPR C (carbon region)) for PCA analysis, for both 1 and 10 mW.

For the two regions, the value inserted is the maximum height of the EPR signal in the ranges, respectively, 3325.3-3385.3 G and 3081.3-3146.3 G. Values are normalized concerning the gain, number of scans, and seed weight, to have comparable results.

From a brief comparison between the EPR spectra, seeds F and G contain higher amounts of copper, while seed E has a different spectrum with low manganese. Little differences are visible among spectrums A-D. We suppose that in the absence of an EPR spectrometer, metal content analysis could give similar results, except for the measurement of carbon radicals.

### High-pressure liquid chromatography

**Fig. 5:**
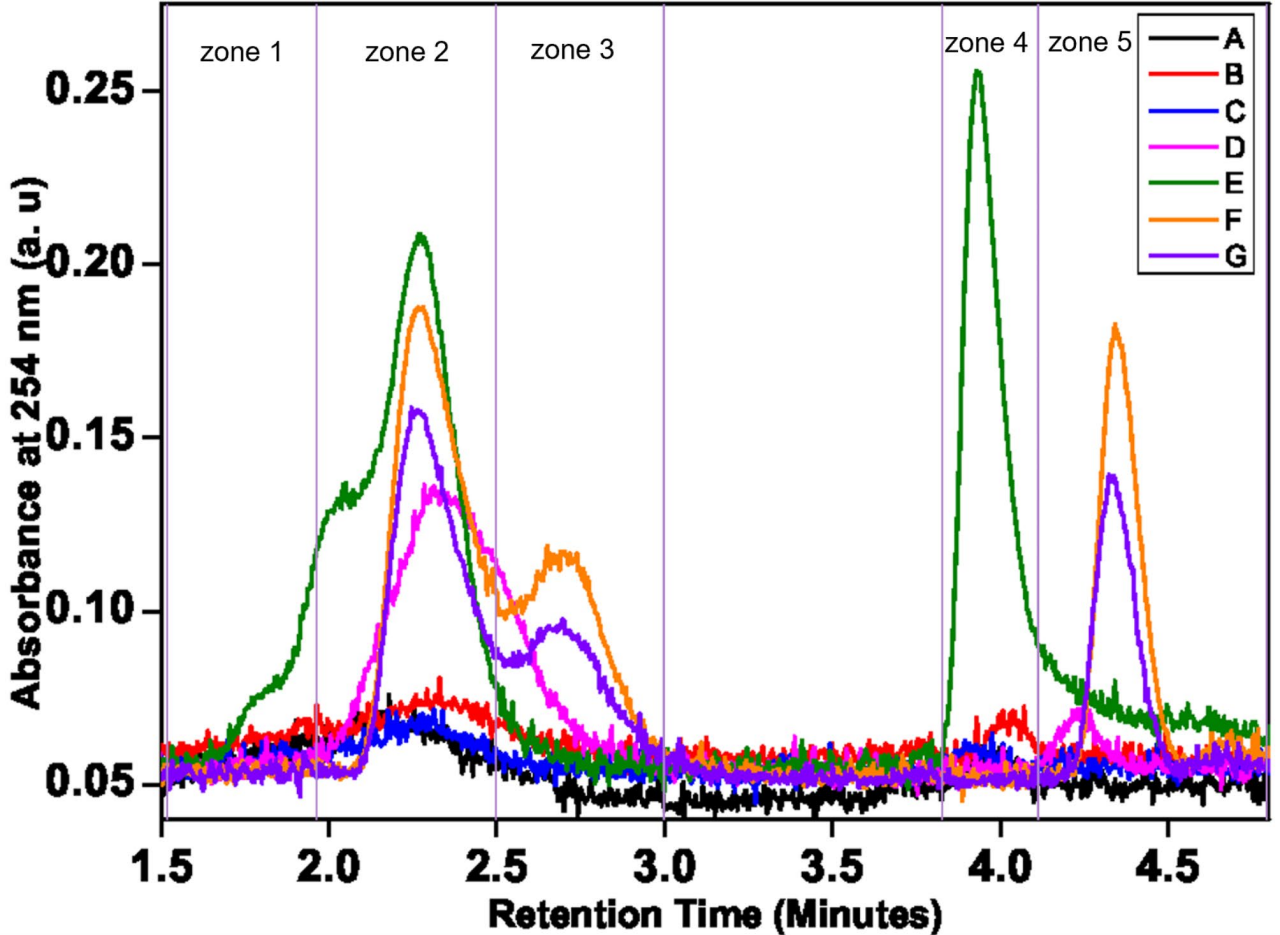
HPLC chromatograms for the sample considered in the paper. Zones are divided into sections or bins, corresponding to the total area, in different regions, named: HPLC zone 1, HPLC zone 2, HPLC zone 3, HPLC zone 4, and HPLC zone 5.

HPLC chromatograms were divided roughly into five zones which are shown in Fig. 5 as indicated in Table 3. The total area of the signal in those zones is considered for the PCA analysis. Since the system is working with a reverse phase column, the more the compounds are polar the less they are retained in the column. Zone 2 corresponds to mixtures of polar compounds like glycosides, phenols, and other polar compounds. Zone 3 is for intermediate polarity secondary metabolites while zones 4 and 5 are for lipids and derivatives.

**Table 3:** Start and end retention times for the integrations of zones defined for PCA.

Observation	HPLC zone 1	HPLC zone 2	HPLC zone 3	HPLC zone 4	HPLC zone 5
Start retention time (min)	1.5	1.95	2.5	3.8	4.1
End retention time (min)	1.95	2.5	3	4.1	4.8

From a brief comparison, seeds F and G represent a group having less polar compounds. Also, seeds E have a different profile at a retention time of 4 min. All the remaining seeds have similar chromatograms, but the D genotype (Jnah Khotifa) exhibits higher polar compounds and secondary metabolites, compared to the three other wheat genotypes. It is worth noting that Jnah Khotifa is an old durum wheat landrace that started to be cultivated in Tunisia in the first half of the 20th century^34^. This landrace was also reported to accumulate higher kernel weight and protein concentration, a relevant trait for the semolina industry^34^. Therefore, we cannot rule out a possible contribution of these polar compounds and secondary metabolites in this specific seed trait.

### Principal component analysis

Principal component analysis (PCA) is a technique used to understand the minimum number of components needed to describe the system under investigation. The technique is a very well-known method used for data analysis and reduction of complexity. We developed our procedure, written in Octave, and released it as an open source (S3). Data coming from observations are placed in a matrix *m* x *n*, where *m* is the number of columns corresponding to different measurements and *n* is the number of samples. In our case, 13 measurements were considered, named: HPLC zone 1, HPLC zone 2, HPLC zone 3, HPLC zone 4, HPLC zone 5, EPR C 1 mW, EPR M 1 mW, EPR C 10 mW, EPR M 10 mW, TGA 25–150, TGA 150–275, TGA 275–350, TGA 350–800 (described in the previous sections).

**Fig. 6:**
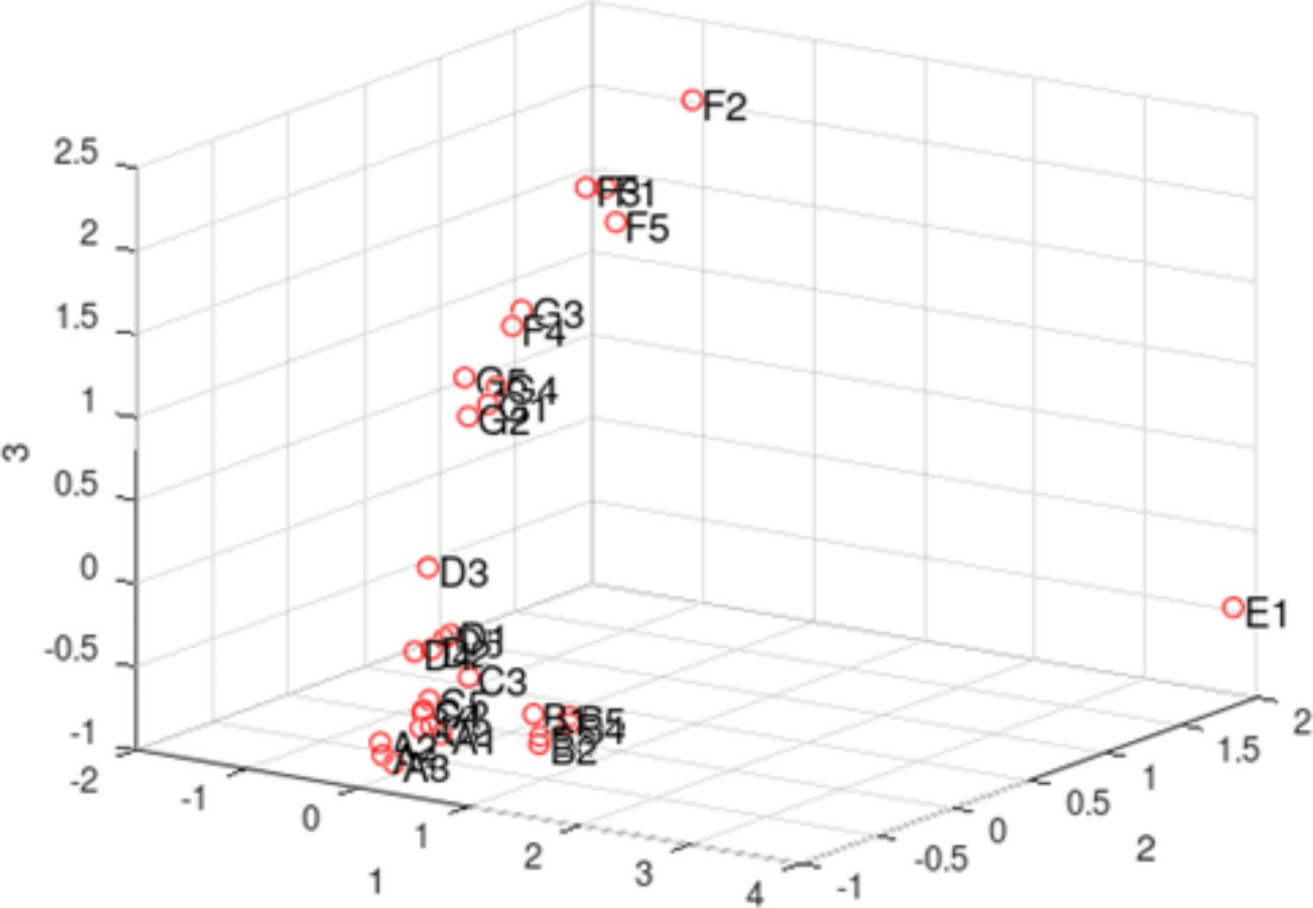
A representation of the reduced space. The number on the axes title represents the number of the component.

First, the matrix is normalized, in a way that all measurements will span the same maximum and minimum values. This is essential to obtain quantitative information from the subsequent analysis that permits performing comparisons between the different columns. PCA transformation can be used to reduce the dimensionality of the observation space. The results of PCA performed by using Octave are shown in Fig. 6. The key transformation is the Single Value Decomposition, from the observation matrix **M**, the decomposition **M** = USV* is created. The most important eigenvectors of **U** give a dimension reduction of the dataset. Data can be shown by using an arbitrary number of eigenvectors to understand similarity. As it is visible from Figure S5, it is sufficient to reduce the space to only one dimension to cluster wheat seeds, while the reduction to 3 dimensions is clearly showing the groups for the different seeds. As it is visible in Fig. 6, seeds A, C, D are in the left-low part of the diagram. Close to this group but in the same region of space are grouped seeds B, while E and F-G are in different volumes of the projection. It is rather unexpected that the seeds B are clustered a bit separately especially from seeds C, knowing that both genotypes Karim and Maali which were introduced relatively recently in Tunisia (in 1982 and 2003, respectively) for their improved yield are phylogenetically related as they share similar pedigrees^35,36^. However, based on several morphological and biochemical traits, they remain distinct. For instance, a significant variability in enzymatic activities such as lipoxygenase and polyphenol oxidase which were reported to play a relevant role in durum wheat flour darkening during pasta processing was observed between Karim and Maali^35,36^.

**Fig. 7:**
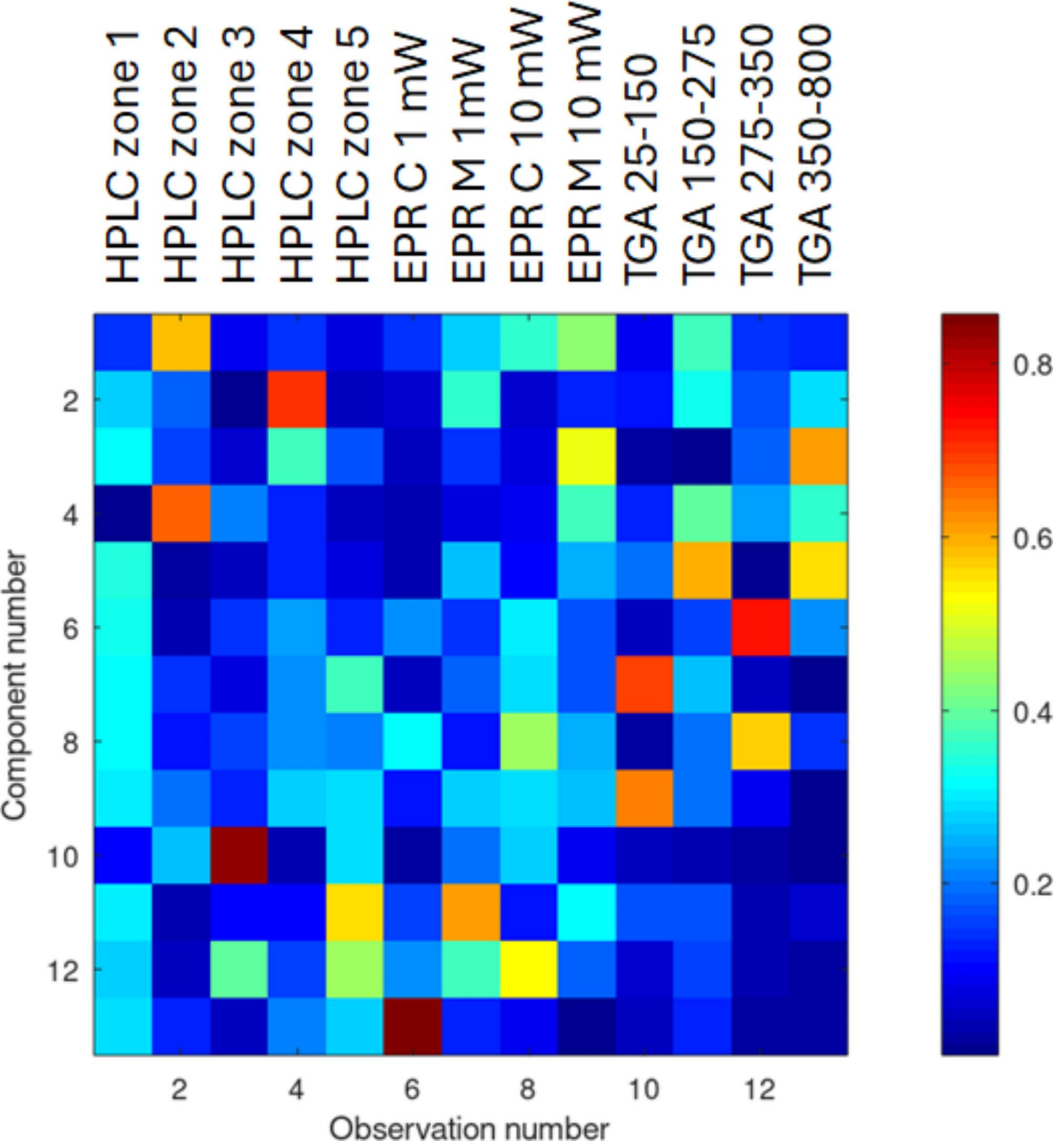
Heatmap corresponds to the representation of the absolute value of **U**, the *m* x *m* matrix coming from PCA. Each row represents a PCA component, starting from the most important (top). Columns are the number of observations, that is the experimental measurements.

For a more comprehensive representation, the projected vectors corresponding to observations are shown in supplementary Figure S7. The matrix **U**, coming from the PCA, is a representation of reduced components of the observation matrix (Fig. 7). In the figure, the columns represent the measurement while the rows are the components. As an example, for the first component, the most important observation is ‘HPLC zone 2’ followed by ‘EPR M 10 mW’. By analyzing the second component, ‘HPLC zone 4’ is the most important observation, in fact, the vector ‘HPLC zone 4’ points in the direction of the maximum variations of the data group (Figure S7). The absolute importance of each component (rows of U) is given by the value of diagonal elements of **S**, shown in Figure S6. Usually, the diagonal components of **S** decrease rapidly, and their magnitude is related to their importance in describing the initial dataset. The analysis of the curve in Figure S6 confirms that the addition of another component contributes negligibly to the description of the initial dataset.

To understand the relative importance of the single analysis groups (HPLC, EPR, and TGA), groups were removed one by one, and PCA analysis was performed again. From that analysis, it was evident that HPLC measurements are playing a key role in the correlation. The removal of all HPLC components gave rise to an unclear classification.

## Conclusions

This research shows that basic physico-chemical characteristics, together with relatively affordable germination analysis can be of importance for the classification of different seeds. Even if a single analytical technique is not sufficient to have a clear classification, the combination of the results by using a simple PCA analysis is determinant. This work aims to create boundaries for more detailed (and complex) techniques with a holistic approach, a fact that is often missing in -omics research. Some of the techniques considered in this paper might be substituted by others more commonly available, such as the use of metal analysis instead of EPR. What is emerging in this work is that HPLC is playing a crucial role in the PCA, because different seeds (from the same species or different species) can have different types of metabolites having different polarity (i.e. oils, phenols or glycosides) and, even if a complete peak assignation is not done, the variation of the relative amount might reveal important differences. It is noteworthy that giving too much importance to the HPLC chromatogram alone might lead to misclassifications, due to the difference in expression of metabolites compared to the environment or the past hystory of the seed. Another important role is given by TGA, where some species have a different profile. Unfortunately, the mean precision of TGA (± 1%) might not be sufficient to make a clear distinction between different seeds, due to the high variability of samples. In our past works we have shown the determination of radicals by using a non-destructive technique as EPR can also have a good correlation with germinability, but this will be the object of further research. In the future, by using simple chemical characterization techniques coupled with principal component analysis (PCA), a holistic technique is used to understand the minimum number of components needed to illustrate the seed specificity using a program Octave (open source).

## Electronic supplementary material

Below is the link to the electronic supplementary material.

Supplementary Material 1

## Data Availability

The authors declare that the data supporting the findings of this study are available within the paper and its Supplementary Information files. Should any raw data files be needed in another format they are available from the corresponding author upon reasonable request. Source data are provided with this paper.
