# Supplementary material for: Exploring seed characteristics and performance through advanced physico-chemical techniques
Source: Sci Rep. 2024 Oct 15;14:24162. doi: 10.1038/s41598-024-75236-0 (PMC11480433; doi:10.1038/s41598-024-75236-0)
Supplement: Supplementary file 1 — Supplementary Material 1 [file 41598_2024_75236_MOESM1_ESM.docx]

**Supplementary document**

**Exploring Seed Characteristics and Performance through Advanced Physico-chemical Techniques**

Dhanalakshmi Vadivel,^1*^ Rania Djemal,^2^ Jessica García,^1^ Andrea Pagano,^3^ Rahma Trabelsi,^4^ Maroua Gdoura-Ben Amor,^4^ Safa Charfeddine,^2^ Siwar Ghanmi,^2^ Ibtisem Khalifa,^4^ Mariem Rekik,^5^ Fatma Amor,^2^ Chantal Ebel,^2^ Radhouane Gdoura,^4^ Amine Elleuch,^5^ Alma Balestrazzi,^3^ Anca Macovei^3^, Moez Hanin^2^ and Daniele Dondi^1^

^1^Department of Chemistry, University of Pavia, Viale Taramelli 12, 27100 Pavia, Italy

^2^Plant Physiology and Functional Genomics Research Unit, Institute of Biotechnology of Sfax, University of Sfax, BP “1175”, Sfax, Tunisia.

^3^Department of Biology and Biotechnology ‘L. Spallanzani', University of Pavia, Via Ferrata 9, 27100 Pavia, Italy.

^4^Research Laboratory of Environmental Toxicology Microbiology and Health (LR17ES06), Faculty of Sciences of Sfax, University of Sfax, BP 1171, Sfax 3000, Tunisia

^5^NCP (National Contact Point) for Horizon Europe Cl6 Food, Bioeconomy, Natural Resources, Agriculture and Environment Laboratory of Plant Biotechnology, Faculty of Science of Sfax, B.P.1171, Sfax 3000, Tunisia

**Sections:**

**1. Thermo gravimetric analysis**

**2. PCA analysis**

**3. Octave code for PCA analysis**

Figures are numbered sequentially.

**1. Thermo gravimetric analysis**

**
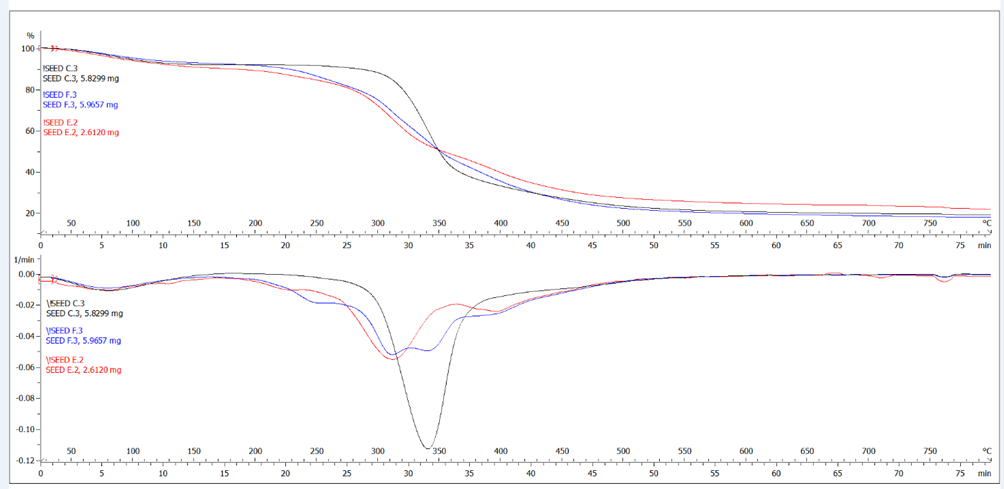
Figure S1.** Comparing different seeds in differential thermal analysis (TGA) as first order derivatives.

Every seed variety is taken into account for the study of differential thermogravimetric analysis to comprehend the TGA. Figure 1 (blue color) illustrates the polymer combination found in Seed F (*Trigonella foenum graecum* L.). The solitary peak in Seed C (*Triticum durum* L.) corresponds to a single polymer. Seed E (*Atriplex halimus* L.), displays the polymer combination.

**
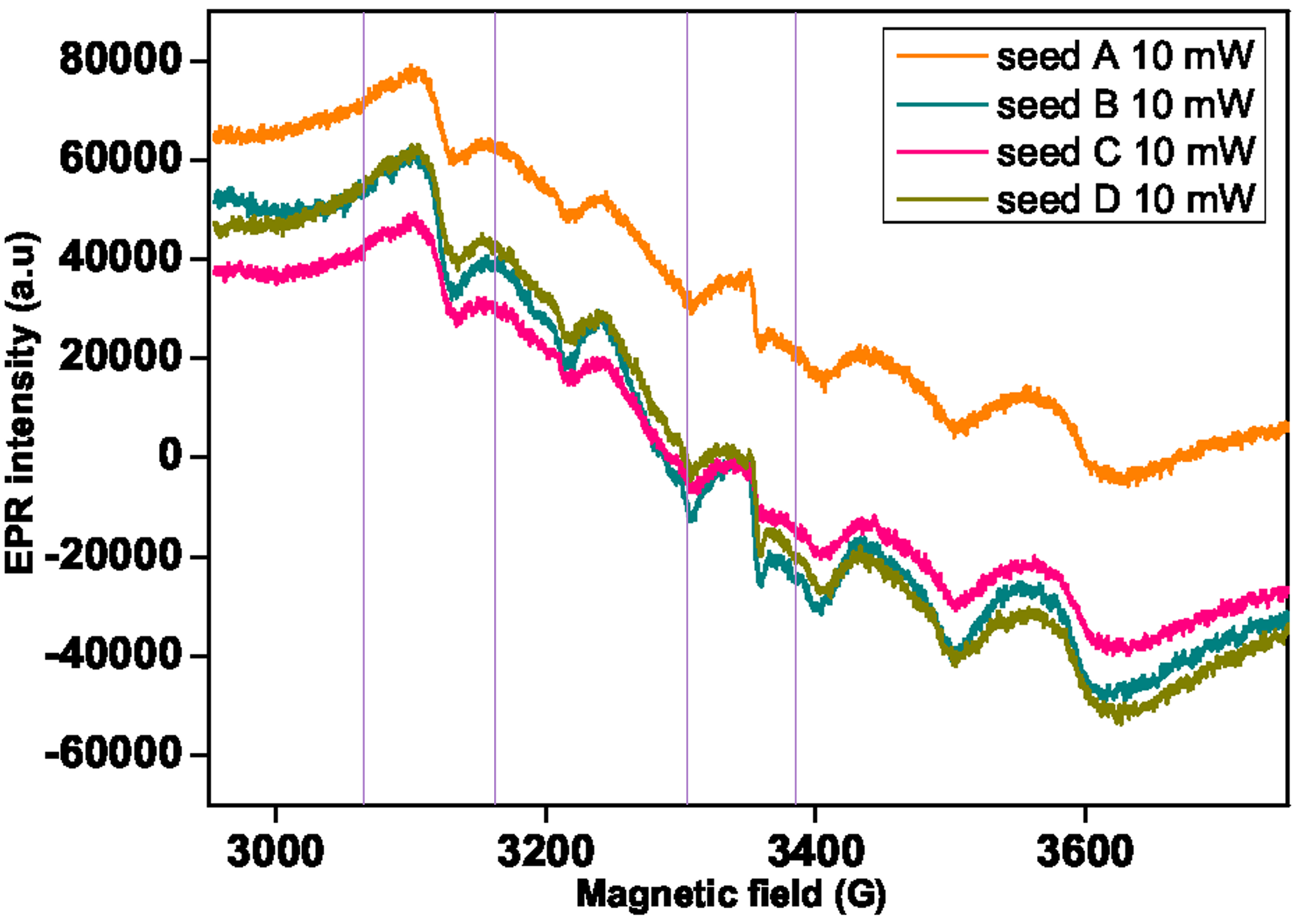
**

**Figure S2.** EPR spectra of the seeds belonging to the wheat (*T. durum* L.) genotypes Mahmoudi (A), Maali (B), Karim (C) and Jneh Khotifa (D) measured with the microwave power of 10 mW at room temperature.

**
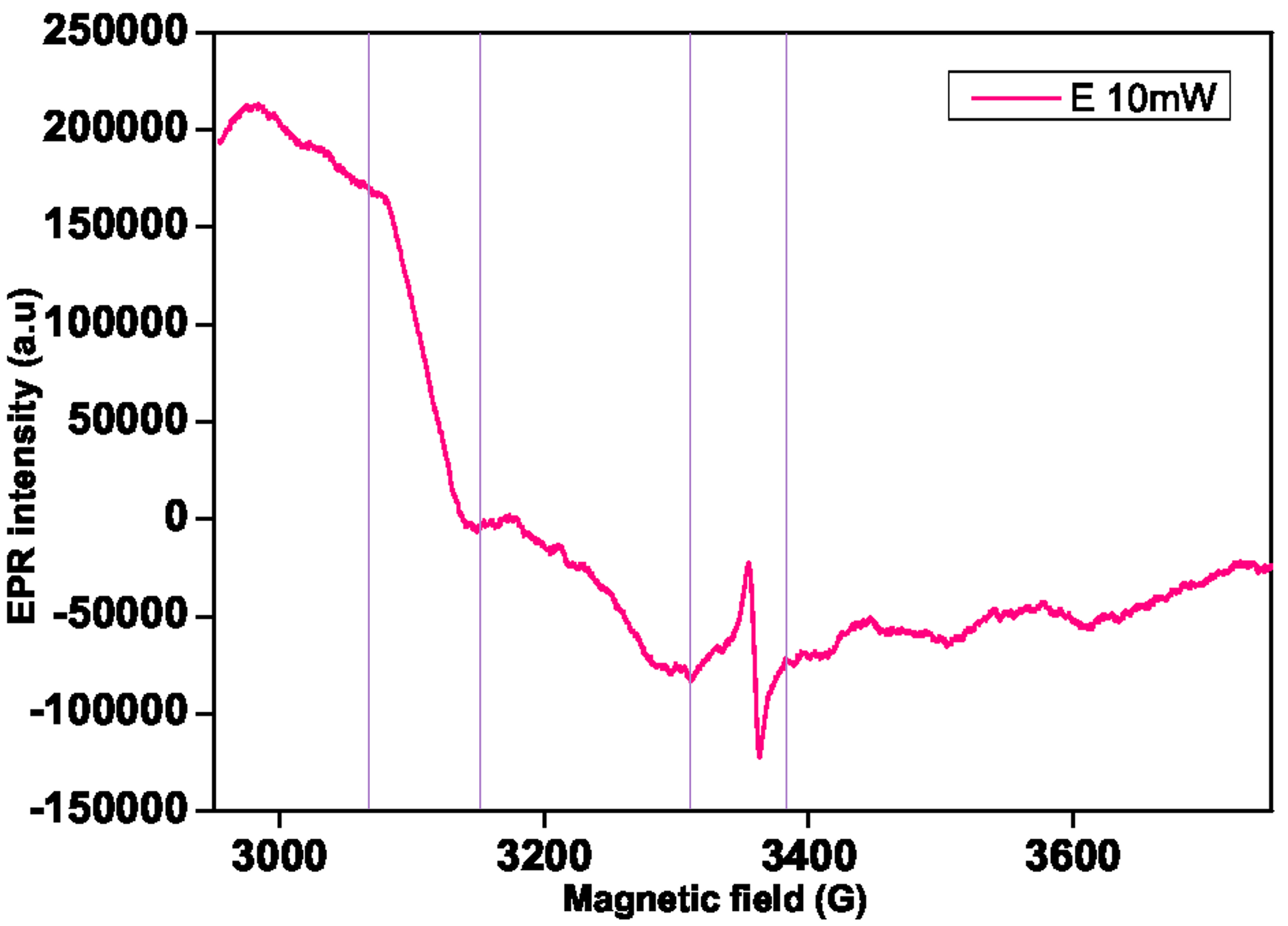
**

**Figure S3.** EPR spectra of the seeds belonging to *Atriplex halimus* L. measured with the microwave power of 10 mW at room temperature.

**
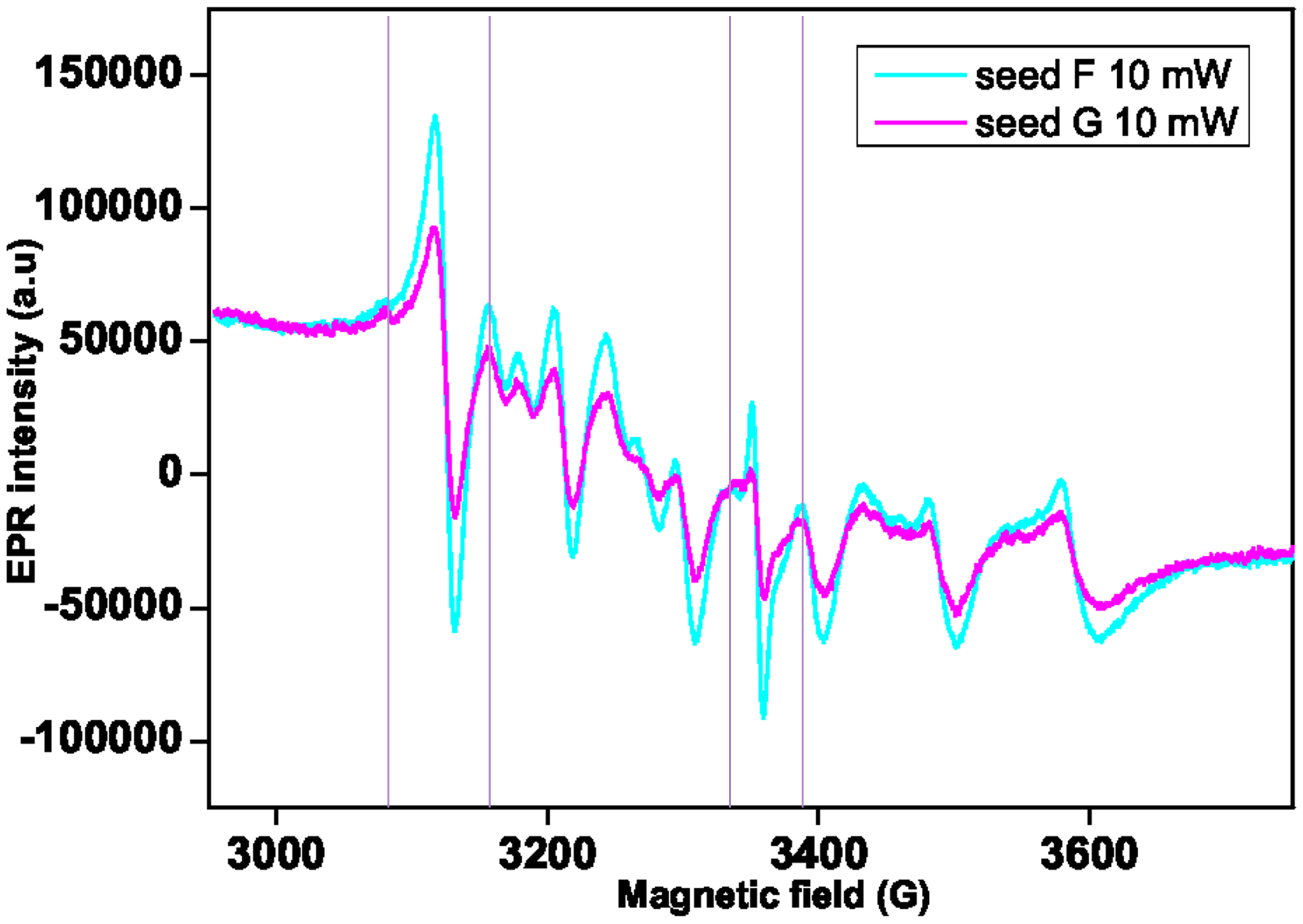
**

**Figure S4.** EPR spectra of seeds belonging to the trigonella (*Trigonella foenum graecum* L.) genotypes F and G measured with the microwave power of 10 mW at room temperature.

**2. PCA analysis**

**
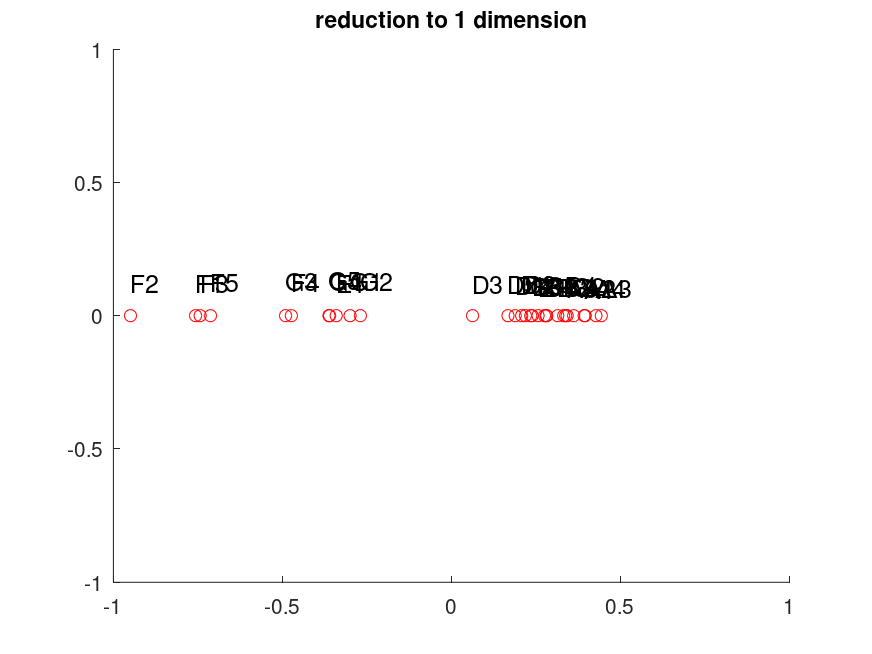
**

**
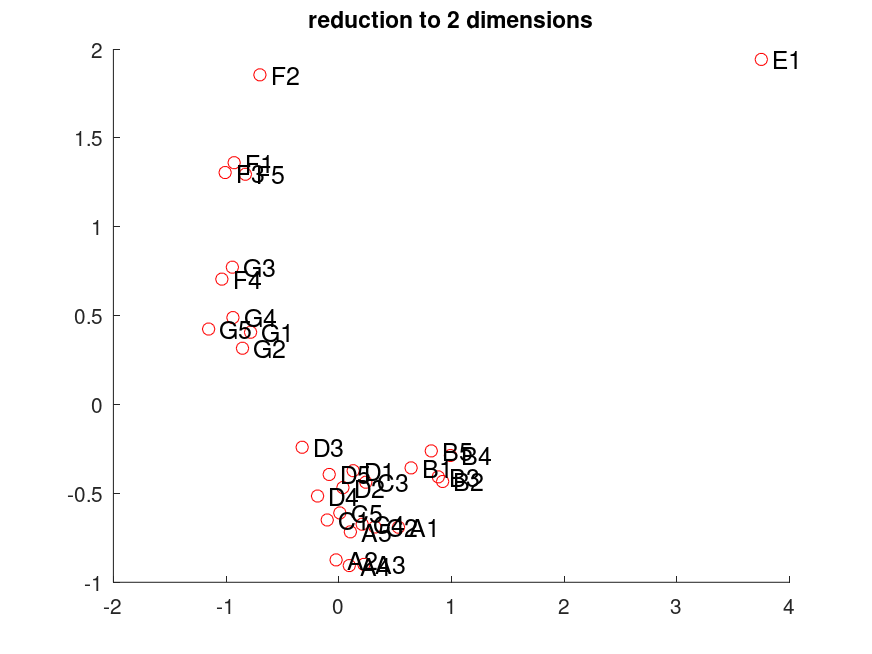

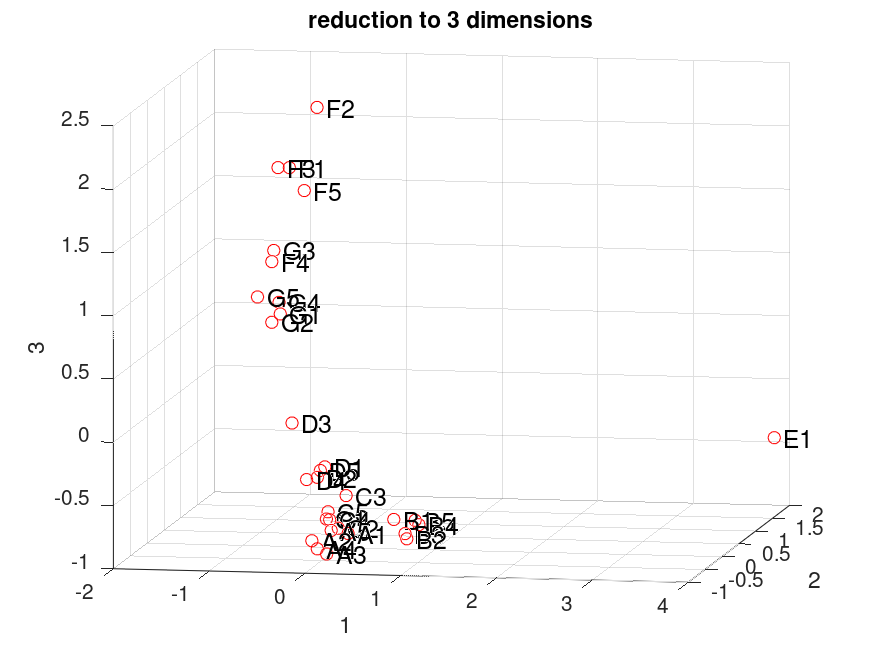
**

**Figure S5.** PCA executed on the dataset in 1, 2, and 3 Dimensions.


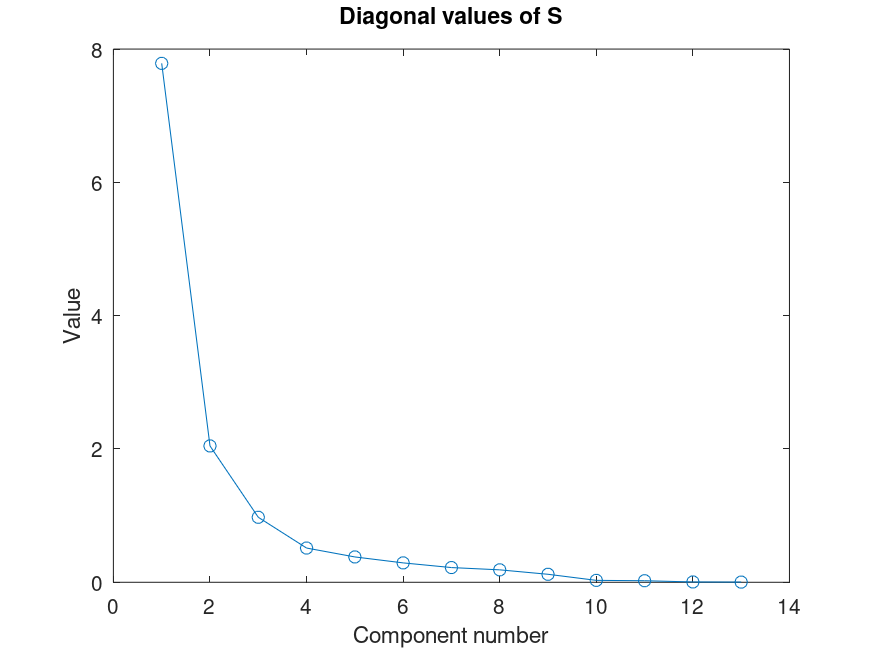


**Figure S6.** Diagonal values of matrix S.


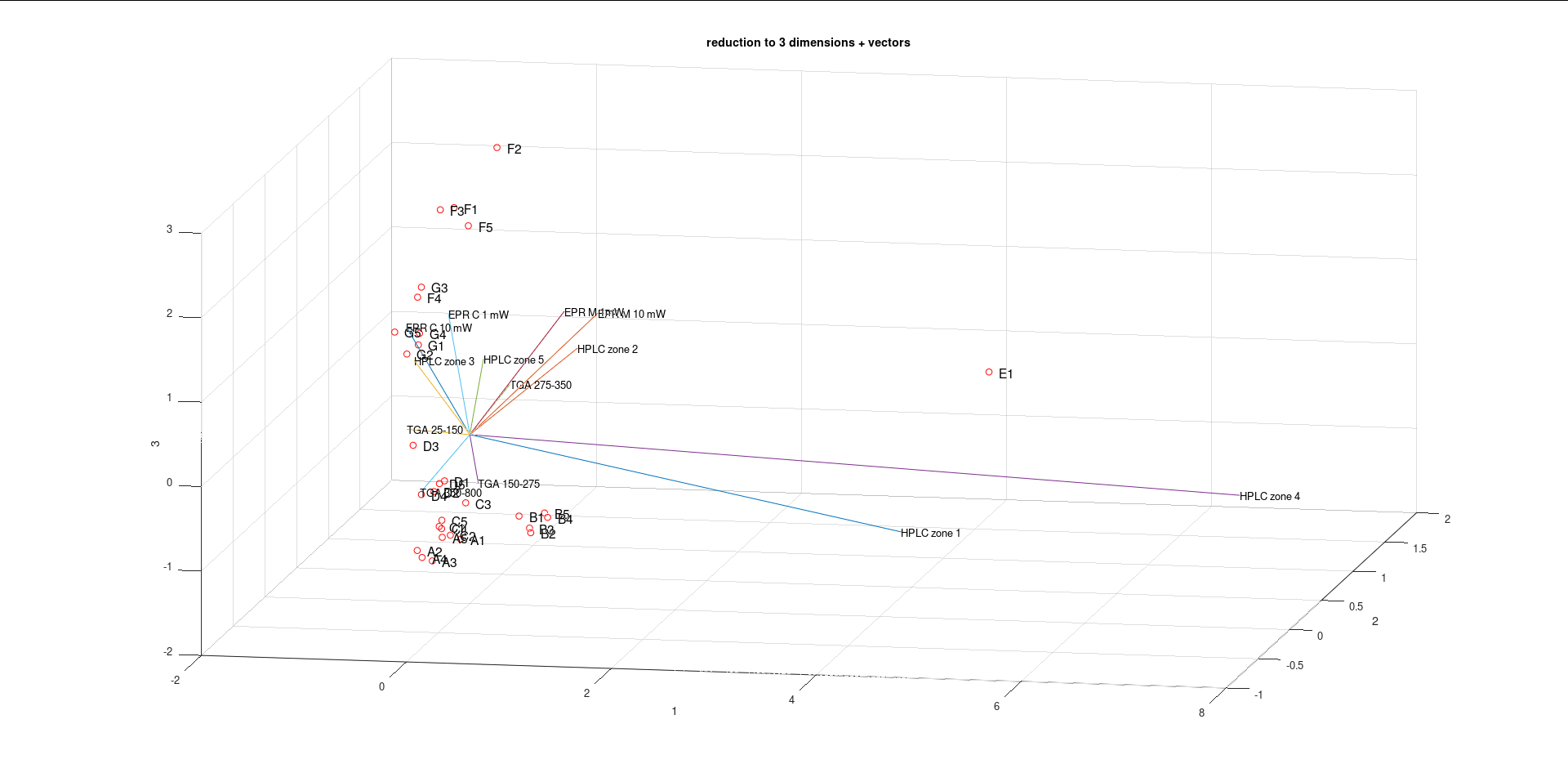


**Figure S7.** Reduction to 3 dimensions with vectors shown.

**3. Octave code for PCA analysis**

Please note that the Octave code is divided into several files:

- Main.m
- drawLine.m
- featureNormalize.m
- pca.m
- projectData.m
- recoverData.m

All files should be in the same folder.

**File Main.m:**

%% Initialization

clear; close all; clc

%% ================== Load Dataset ===================

% The following command loads the dataset.

% all data should be listed in an ASCII text file separated by commas

% the first row of the file should be column names

% the first column of the file should be the sample name/code

Y=importdata('data.csv');

X=Y.data;

[NumRow NumCol]=size(X);

labels=Y.rowheaders(:,1)

col_names=strsplit(char(Y.textdata(1,1)),',')(2:end)

file_path = fileparts(mfilename('fullpath'));

% Before running PCA, it is important to first normalize X

% mu is the mean calculated column wise

% sigma is the standard deviation calculated column wise

[X_norm, mu, sigma] = featureNormalize(X);

figure_num=0;

% Visualize the example dataset choosing the columns having the maximum median

[a b]=sort(abs(median(X_norm)),"descend"); %in b the indices of the columns having the maximum median, ordered from the highest

figure_num+=1;

h=figure(figure_num);

plot3(X(:, b(1)), X(:, b(2)), X(:, b(3)) ,'bo');

title("plot with respect to most important medians")

xlabel(['1st ' col_names(b(1))])

ylabel(['2nd ' col_names(b(2))])

zlabel(['3rd ' col_names(b(3))])

hold on;

for i = 1:size(X, 1)

text(X(i, b(1))+0.1, X(i, b(2)), X(i, b(3)), labels(i),'FontSize', 12);

end

hold off

saveas(h,fullfile(file_path,strcat('figure',int2str(figure_num),'.png')));

%% =============== Principal Component Analysis ===============

%

% Run PCA

[U, S] = pca(X_norm);

% Draw the eigenvectors centered at mean of data. These lines show the

% directions of maximum variations in the dataset.

hold on;

drawLine(mu, mu + 1.5 * S(1,1) * U(:,1)', '-k', 'LineWidth', 2);

drawLine(mu, mu + 1.5 * S(2,2) * U(:,2)', '-k', 'LineWidth', 2);

drawLine(mu, mu + 1.5 * S(3,3) * U(:,3)', '-k', 'LineWidth', 2);

hold off;

saveas(h,fullfile(file_path,strcat('figure',int2str(figure_num),'B.png')));

fprintf('Top 3 eigenvectors: \n');

U(1,:)

U(2,:)

U(3,:)

[a b]=sort(abs(U(1,:)),"descend");

fprintf('Top 3 components of first eigenvector: \n');

col_names(b(1:3))

U

diag(S)

%% =================== Plot abs(U) as heatmap ===================

figure_num+=1;

h=figure(figure_num);

colormap('jet');

imagesc(abs(U));

colorbar;

xlabel('Observation number');

ylabel('Component number');

saveas(h,fullfile(file_path,strcat('figure',int2str(figure_num), ' U heatmap','.png')));

%% =================== Plot diag(S) ===================

figure_num+=1;

h=figure(figure_num);

plot(diag(S),'marker','o');

title([ "Diagonal values of S"])

xlabel('Component number');

ylabel('Value');

saveas(h,fullfile(file_path,strcat('figure',int2str(figure_num),' S','.png')));

%% =================== 1 Dimension Reduction ===================

%

% Plot the normalized dataset (returned from pca)

figure_num+=1;

h=figure(figure_num);

% Project the data onto K dimensions

K = 1;

title([ "reduction to " int2str(K) " dimension"])

Z = projectData(X_norm, U, K);

X_rec = recoverData(Z, U, K);

hold on;

plot(X_rec(:, 1), 0,'ro');

for i = 1:size(X_norm, 1)

text(X_rec(i,1), 0.1+i*0.001, labels(i),'FontSize', 12);

end

hold off

saveas(h,fullfile(file_path,strcat('figure',int2str(figure_num),' 1D.png')));

%% =================== 2 Dimensions Reduction ===================

%

% Plot the normalized dataset (returned from pca)

figure_num+=1;

h=figure(figure_num);

% Project the data onto K dimensions

K = 2;

title([ "reduction to " int2str(K) " dimensions"])

Z = projectData(X_norm, U, K);

X_rec = recoverData(Z, U, K);

hold on;

plot(X_rec(:, 1), X_rec(:, 2),'ro');

for i = 1:size(X_norm, 1)

text(X_rec(i,1)+0.1, X_rec(i,2), labels(i),'FontSize', 12);

end

hold off

saveas(h,fullfile(file_path,strcat('figure',int2str(figure_num),' 2D.png')));

%% =================== 3 Dimensions Reduction ===================

%

% Plot the normalized dataset (returned from pca)

figure_num+=1;

h=figure(figure_num);

% Project the data onto K dimensions

K = 3;

title([ "reduction to " int2str(K) " dimensions"])

Z = projectData(X_norm, U, K);

X_rec = recoverData(Z, U, K);

hold on;

xlabel('1');

ylabel('2');

zlabel('3');

plot3(X_rec(:, 1), X_rec(:, 2), X_rec(:, 3),'ro');

view(10,10)

grid on

for i = 1:size(X_norm, 1)

text(X_rec(i,1)+0.1, X_rec(i,2), X_rec(i,3),labels(i),'FontSize', 12);

end

saveas(h,fullfile(file_path,strcat('figure',int2str(figure_num),' 3D.png')));

% ===================== 3 dimensions + vectors =====================

figure_num+=1;

h=figure(figure_num);

% Project the data onto K dimensions

K = 3;

title([ "reduction to " int2str(K) " dimensions + vectors"])

Z = projectData(X_norm, U, K);

X_rec = recoverData(Z, U, K);

hold on;

xlabel('1');

ylabel('2');

zlabel('3');

plot3(X_rec(:, 1), X_rec(:, 2), X_rec(:, 3),'ro');

view(10,10)

grid on

for i = 1:size(X_norm, 1)

text(X_rec(i,1)+0.1, X_rec(i,2), X_rec(i,3),labels(i),'FontSize', 12);

end

%plots the vectors

Origin = [0,0,0];

for property=1:NumCol

versor=zeros(1,NumCol);

versor(property)=max(X_norm(:,property))*3; %S(property,property)*3;

Z = projectData(versor, U, K);

top_eigen=recoverData(Z, U, K);

text(top_eigen(1), top_eigen(2), top_eigen(3),col_names(property),'FontSize', 10);

pts = [Origin; top_eigen(1:3)];

plot3(pts(:,1), pts(:,2), pts(:,3)); %,'Color','red','LineWidth',1);

end

hold off

saveas(h,fullfile(file_path,strcat('figure',int2str(figure_num),' 3D plus Vectors.png')));

fprintf('Program finished.\n');

**File drawLine.m:**

function drawLine(p1, p2, varargin)

%DRAWLINE Draws a line in 3D from point p1 to point p2

% DRAWLINE(p1, p2) Draws a line from point p1 to point p2 and holds the

% current figure

plot3([p1(1) p2(1)], [p1(2) p2(2)], [p1(3) p2(3)], varargin{:});

End

**File featureNormalize.m:**

function [X_norm, mu, sigma] = featureNormalize(X)

%FEATURENORMALIZE Normalizes the features in X

% FEATURENORMALIZE(X) returns a normalized version of X where

% the mean value of each feature is 0 and the standard deviation

% is 1. This is often a good preprocessing step to do when

% working with learning algorithms.

mu = mean(X);

X_norm = bsxfun(@minus, X, mu);

sigma = std(X_norm);

X_norm = bsxfun(@rdivide, X_norm, sigma);

% ============================================================

end

**File pca.m:**

function [U, S] = pca(X)

%PCA Run principal component analysis on the dataset X

% [U, S, X] = pca(X) computes eigenvectors of the covariance matrix of X

% Returns the eigenvectors U, the eigenvalues (on diagonal) in S

%

% Useful values

[m, n] = size(X);

U = zeros(n);

S = zeros(n);

Sigma = 1/m*(X'*X);

[U,S,V] = svd(Sigma);

% =========================================================================

end

**File projectData.m:**

function Z = projectData(X, U, K)

%PROJECTDATA Computes the reduced data representation when projecting only

%on to the top k eigenvectors

% Z = projectData(X, U, K) computes the projection of

% the normalized inputs X into the reduced dimensional space spanned by

% the first K columns of U. It returns the projected examples in Z.

%

Z = zeros(size(X, 1), K);

Ureduce = U(:,1:K);

Z = X*Ureduce;

% =============================================================

end

**File recoverData.m:**

function X_rec = recoverData(Z, U, K)

%RECOVERDATA Recovers an approximation of the original data when using the

%projected data

% X_rec = RECOVERDATA(Z, U, K) recovers an approximation the

% original data that has been reduced to K dimensions. It returns the

% approximate reconstruction in X_rec.

%

X_rec = zeros(size(Z, 1), size(U, 1));

% ====================== YOUR CODE HERE ======================

% Instructions: Compute the approximation of the data by projecting back

% onto the original space using the top K eigenvectors in U.

%

% For the i-th example Z(i,:), the (approximate)

% recovered data for dimension j is given as follows:

% v = Z(i, :)';

% recovered_j = v' * U(j, 1:K)';

%

% Notice that U(j, 1:K) is a row vector.

%

Ureduce = U(:,1:K);

X_rec = (Ureduce*Z')';

% =============================================================

end
